# A comparison of isomaltulose versus maltodextrin ingestion during soccer-specific exercise

**DOI:** 10.1007/s00421-017-3719-5

**Published:** 2017-09-19

**Authors:** Emma J. Stevenson, Anthony Watson, Stephan Theis, Anja Holz, Liam D. Harper, Mark Russell

**Affiliations:** 1 Institute of Cellular Medicine, Newcastle University, Newcastle upon Tyne, UK; 2 Human Nutrition Research Centre, School of Agriculture, Food and Rural Development, Newcastle University, Newcastle upon Tyne, UK; 3 BENEO-Institute, Obrigheim/Pfalz, Wormserstrasse 11, Obrigheim, 67283 Germany; 4 Human and Health Sciences, University of Huddersfield, Huddersfield, UK; 5 School of Social and Health Sciences, Leeds Trinity University, Leeds, LS18 5HD UK

**Keywords:** Extra-time, Football, Half-time, Carbohydrate, Rebound hypoglycaemia

## Abstract

**Purpose:**

The performance and physiological effects of isomaltulose and maltodextrin consumed intermittently during prolonged soccer-specific exercise were investigated.

**Methods:**

University soccer players (*n* = 22) performed 120 min of intermittent exercise while consuming 8% carbohydrate–electrolyte drinks (equivalent to ~ 20 g h^−1^) containing maltodextrin (Glycaemic Index: 90–100), isomaltulose (Glycaemic Index: 32) or a carbohydrate-energy-free placebo in a manner replicating the practices of soccer players (i.e., during warm-up and half-time). Physical (sprinting, jumping) and technical (shooting, dribbling) performance was assessed.

**Results:**

Blood glucose and plasma insulin (both *P* < 0.001) concentrations varied by trial with isomaltulose maintaining > 13% higher blood glucose concentrations between 75 and 90 min versus maltodextrin (*P* < 0.05). A decline in glycaemia at 60 min in maltodextrin was attenuated with isomaltulose (−19 versus −4%; *P* = 0.015). Carbohydrates attenuated elevations in plasma epinephrine concentrations (*P* < 0.05), but isomaltulose proved most effective at 90 and 120 min. Carbohydrates did not attenuate IL-6 increases or reductions in physical or technical performances (all *P* > 0.05). Ratings of abdominal discomfort were influenced by trial (*P* < 0.05) with lower values for both carbohydrates compared to PLA from 60 min onwards.

**Conclusions:**

Although carbohydrates (~ 20 g h^−1^) did not attenuate performance reductions throughout prolonged soccer-specific exercise, isomaltulose maintained higher blood glucose at 75–90 min, lessened the magnitude of the exercise-induced rebound glycaemic response and attenuated epinephrine increases whilst maintaining similar abdominal discomfort values relative to maltodextrin. When limited opportunities exist to consume carbohydrates on competition-day, low-glycaemic isomaltulose may offer an alternative nutritional strategy for exercising soccer players.

## Introduction

Soccer is a high-intensity intermittent team sport that typically requires players to complete 90 min of activity separated by a half-time (HT) break; however, when scores are equal at 90 min and an outright winner is required, extra-time is played. Between 1998 and 2014, approximately 35% of knockout phase matches played in FIFA World Cup competitions have required the 30-min extra-time period (http://www.FIFA.com). Physical and skilled performance reduces throughout soccer-specific exercise of 90- (Harper et al. 2017; Russell et al. 2011a, 2012) and 120-min durations (Harper et al. 2014, 2016a, b). Therefore, interventions that maintain performance throughout the full duration of match-play are likely of interest when seeking to optimise the practices of soccer players.

Carbohydrate–electrolyte beverages are often recommended to soccer players before and during exercise in order to maintain glycaemia and fuel provision, and attenuate the risk of dehydration (Convertino et al. 1996; Thomas et al. 2016). Indeed, ergogenic effects have been observed following the regular ingestion (i.e., every 15 min) of carbohydrates throughout 90 min of simulated match-play (Kingsley et al. 2014; Russell et al. 2012). However, regular fluid ingestion does not align with applied practices as professional players may only consume fluids during scheduled breaks in play (e.g., HT). The metabolic response to ~ 68 g of total carbohydrates (7 ml kg^−1^ body mass) consumed during 90 min of intermittent exercise does not seem to differ when ingested over two or six ingestion points (Clarke et al. 2008); however, owing to an omission of performance measures by Clarke et al. (2008), limited information exists to ascertain whether performance as opposed to metabolic responses are mediated by different feeding strategies.

Commercially available sports beverages generally consist of 6–10% concentrations of carbohydrates, of which high-glycaemic index (GI) carbohydrate sources such as maltodextrin are common constituents. Paradoxically, high-GI carbohydrates consumed within an hour of commencing a single bout of exercise may elicit rebound hypoglycaemia 15–30 min into subsequent activity (Chryssanthopoulos et al. 1994; Costill et al. 1977); a response for which the mechanisms, and performance consequences, are currently unclear (Jentjens and Jeukendrup 2003). In both simulated and actual soccer match-play, carbohydrate–electrolyte beverages ingested every 15 min during exercise and before each half, have also been shown to elicit a transient reduction in glycaemia at 45–60 min (Kingsley et al. 2014; Russell et al. 2012, 2014). Notably, Kingsley et al. (2014) observed hypoglycaemia (i.e., defined as < 3.8 mmol l^−1^) in > 50% of participants consuming carbohydrate beverages throughout simulated soccer match-play. Given the ergogenic effects of carbohydrates that maintain glycaemia throughout intermittent exercise (Harper et al. 2016a, 2017; Russell et al. 2012; Russell and Kingsley 2014), optimisation of match-day nutritional strategies is desirable for soccer players (Russell et al. 2015c).

The GI of the ingested carbohydrate appears to influence the occurrence of the rebound hypoglycaemic response at the onset of a single exercise bout (Jentjens and Jeukendrup 2003); possibly attributable to the degree of hyperinsulinemia observed post-ingestion (Costill et al. 1977). Isomaltulose is a disaccharide comprised of a glucose and fructose unit joined by an α-1,6-glycosidic bond which is less readily hydrolysed by digestive enzymes in the human gastrointestinal tract when compared to the α-1,4-linked monosaccharide units in maltodextrin. As a consequence of slower rates of hydrolysis (Dahlqvist et al. 1963) and subsequent absorption at the intestinal mucosa (MacDonald and Daniel 1983), isomaltulose results in a low-glycaemic and low-insulinemic response and prolongs the delivery of glucose to the systemic circulation. While it is acknowledged that prolonged versus expedited appearance of exogenous energy consumed in substantial amounts (i.e., > 60 g h^−1^) may affect gastric comfort and performance during exercise (Oosthuyse et al. 2015), such practices do not replicate the fluid-intake strategies of team sports players. Notably, fluid intake during competitive soccer may only occur during scheduled breaks in play (e.g., HT) and in the final stages of the warm-up and the immediate period before kick-off (Harper et al. 2017). Thus, fluid intake seldom occurs as often as has previously been examined (i.e., every 15 min).

As no studies have investigated the effects of isomaltulose during soccer-specific exercise, and there is evidence that low-GI carbohydrates reduce the incidence of hypoglycaemic episodes when investigating the occurrence of rebound hypoglycaemia (Jentjens and Jeukendrup 2003), the aim of this study was to examine the performance and physiological effects of low-GI isomaltulose and high-GI maltodextrin consumed before and during 120 min of simulated soccer match-play using fluid intake patterns that better represent the demands of competition. The hypothesis associated with this study was that isomaltulose would prolong blood glucose availability and thereby influence performance and physiological responses during soccer-specific exercise.

## Methods

### Participants

Following ethical approval for human studies (in accordance with the ethical standards laid down in the 1964 Declaration of Helsinki and its later amendments) and written informed consent being attained, 22 male university standard soccer players (age: 20 ± 2 years, mass: 73.0 ± 7.9 kg, stature: 1.79 ± 0.07 m, estimated $\dot {V}{\text{O}_2}$_max_: 54.8 ± 3.1 ml kg^−1^ min^−1^) with > 1 year’s playing experience completed the study. Retrospective power analyses highlighted that the sample size was adequate for > 80% statistical power when detecting differences between physiological responses. Exclusion criteria included diagnosis of any significant medical condition or disorder that may have interfered with participation (e.g., cardiovascular disease, musculoskeletal disorders, diabetes mellitus).

### Experimental procedures

All participants completed two preliminary visits to the laboratory followed by three main trials (Maltodextrin: MDX, high-GI; PALATINOSE™: PSE, low-GI; placebo: PLA, no carbohydrates). Each player was advised to refrain from strenuous physical activity and caffeine consumption during the 72 h preceding all testing sessions and dietary intake was recorded for 48 h before each main trial (retrospectively analysed with: MicroDiet; Downlee Systems Ltd., High Peak, UK). A standardised evening meal was consumed on the night before each main trial (Energy content: 3.3 MJ, 92 g carbohydrate, 28 g fat and 37 g protein; representing 0.04–0.06 MJ kg^−1^ body mass).

### Preliminary testing

Following arrival at the first preliminary testing session, players voided their bowels and bladder before body mass (model 876; Seca GmbH, Hamburg, Germany) and stature (Portable Stadiometer; Holtain Ltd, Wales, UK) were measured. A controlled warm up (~ 20-min), including dynamic stretches and movements that progressed from low to moderate intensity, was then performed. Soccer skill practice (~ 5 min) preceded performance of four 20-m sprints (interspersed with 45 s active recovery) that progressed to near-maximal speeds. Thereafter, maximal oxygen uptake was estimated (Ramsbottom et al. 1988) with a minimum value of 48.5 ml kg^−1^ min^−1^ required to allow completion of the 120-min protocol. The second preliminary session habituated participants with the testing procedures of the main trials.

### Main experimental trials

Players attended the laboratory at 08:15 h following an overnight fast. Upon arrival, a mid-flow urine sample was provided and urine osmolality was measured (Model 3300 Micro-Osmometer; Advanced Instruments Inc., Norwood, MA, USA). Thereafter, a resting venous blood sample was taken before a standardised breakfast providing ~ 10% of the individual’s daily energy requirement (Rice Krispies; Kellogg’s, UK, semi-skimmed milk and 500 ml of mineral water; 1.23 ± 0.08 MJ, consisting of 52.0 ± 3.3 g carbohydrate, 4.7 ± 0.3 g fats and 10.9 ± 0.7 g proteins) was consumed. Body mass and stature were then measured prior to ~ 90 min of rest; upon which a pre-exercise blood sample was taken. Players then commenced their final preparations before performing the same standardised warm-up as used in the preliminary testing. A 5-min period of passive recovery followed the end of the warm-up.

Measurements of physical and skilled performance preceded the start of exercise. Participants then performed 120 min of exercise and skills testing, consisting of two 45-min halves and two further 15-min periods (extra-time) of simulated match-play using a modified version of the Soccer Match Simulation (SMS; Russell et al. 2011b). Post-exercise assessments of performance, hydration status and body mass preceded a standardised cool down. All testing was performed indoors and environmental conditions were similar between trials (all *P* > 0.05; temperature: 18.2 ± 0.8 °C, pressure: 1033 ± 25 mmHg, humidity: 38 ± 4%).

#### Soccer match simulation

**Fig. 1:**
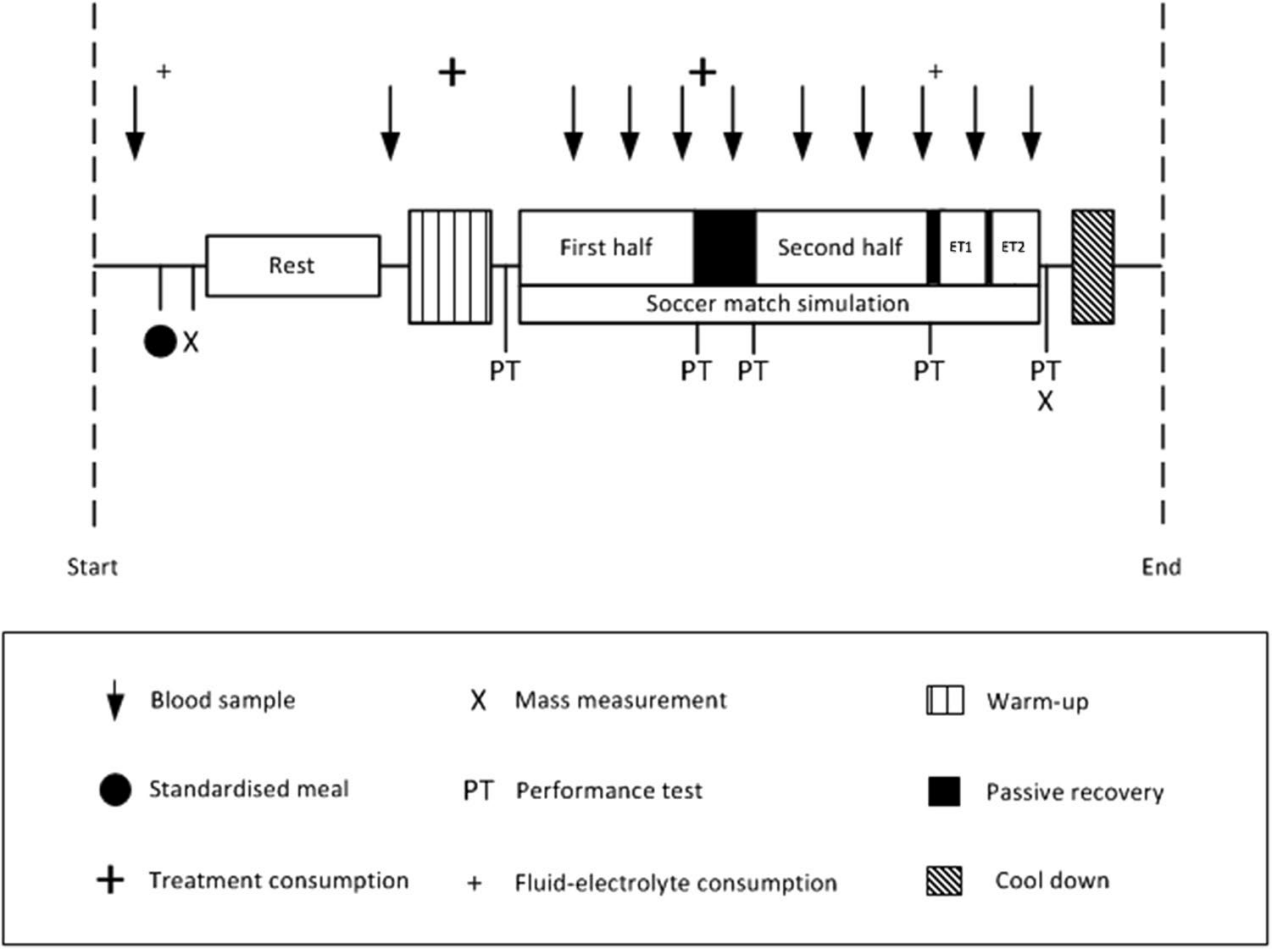
Overview of main trial procedures

The modified version of the SMS required players to cover ~ 14.4 km (reflecting matches requiring extra-time; Russell et al. 2015a) at various running intensities, with backward and sideward movements over a 20-m distance, while intermittently performing 15-m sprints and soccer dribbling. Compared to the original SMS (Russell et al. 2011b), the assessment of soccer passing was omitted (such that more invasive blood samples could be taken) and extra-time was required. In line with UEFA playing regulations, a 15-min passive recovery period (HT) separated the two 45-min halves, whereas a 2-min passive recovery period separated each extra-time half and a 5-min rest period preceded extra-time (Fig. 1). The repeatability of the responses to 120 min of the original SMS has been determined (Harper et al. 2016b).

#### Performance testing

Countermovement jump height, repeated sprint ability and soccer shooting performance were assessed on five occasions (performance test, PT) throughout each trial (i.e., Pre 1st half, post 1st half, pre 2nd half, Post 2nd half, post extra-time; Fig. 1), whereas dribbling and 15 m sprint velocity were assessed throughout the SMS. Countermovement jump height was determined using an electronic system (OptoJump Next; Microgate SRL, Bolzano, Italy). Participants performed three repetitions (with 10 s of passive recovery) with the peak value used for subsequent analyses. Timed 15 m sprint performance (Brower Timing Systems, Utah, USA) was assessed throughout the SMS and values represent means per 15 min of exercise.

Repeated sprint maintenance was assessed from a standing start (from 0.3 m behind the first timing gate) and participants performed three maximal 20 m sprints, separated by 25 s of active recovery. The peak sprint velocity achieved during these three attempts was used to derive a sprint decrement index from the percentagewise reduction of sprint velocity between the slowest and fastest sprints.

Soccer shooting performance was assessed using methods similar to Russell et al. (2010). Balls (Mitre Promax: size 5; Mitre Sports International, London, England) released from behind participants towards a 1.5 × 1.5-m action zone, were required to be kicked towards one of four randomly illuminating targets placed in the corners of a standard adult soccer goal (7.32 × 2.44-m). The shooting assessments consisted of four attempts and participants were instructed to use the foot that they felt was most suitable to complete the task. The dribbling test has been described previously (Russell et al. 2010) and required participants to dribble a ball over a 20-m track as fast and as accurately as possible. The reliability and validity of these tests have been reported (Russell et al. 2010).

Skilled performance was analysed according to Russell et al. (2010) from 50 Hz video footage (DCR-HC96E; Sony Ltd, UK). Briefly, three outcome measures were calculated for each skill, including: precision (distance from target), percentage success and average ball speed. Precision was determined by digitising video footage (Kinovea version 0.8.15; Kinovea Org., France) to determine the distance of the centre of the ball from the centre of the target. Success in shooting was defined by shots taken within the confines of the action zone and where the ball impacted within the goal area. During dribbling, if a cone was touched by the ball or was not completed in the required direction, the cone was considered to be unsuccessfully negotiated. Dribbling success was defined as the percentage of cones negotiated successfully. Average ball speeds were calculated for all skills (Kinovea version 0.8.15; Kinovea Org., France) and shooting variables are expressed as an average at each time-point, whereas measures of dribbling performance are expressed per 15 min of exercise.

#### Physiological testing

Venous blood samples were taken from a cannula that was inserted in the cephalic vein (20 gauge; BD Venflon, BD, New Jersey, USA). While participants were in a recumbent position, venous blood was drawn at rest, pre-exercise (Pre), half-time (HT; 10 min into rest period) and every 15 min during exercise. Cannula patency was maintained by saline infusion (~ 5 ml). Blood was collected in two 6-ml vacutainers (EDTA and Lipid Heparin) and a 2-ml luer at each time-point. Vacutainers were centrifuged at 3000 revolutions min^−1^ for 10 min at 4 °C (Allegra X-22R; Beckman Coulter Ltd., California, USA) with the resultant plasma subsequently frozen at −80 °C. Blood from the luer was used to determine glucose and lactate concentrations (Biosen C-Line Clinic; EKF Diagnostics, Cardiff, UK).

Plasma volume changes were calculated at each time-point by measurement of haemoglobin (Hemocue Hb 201 + System; Hemocue AB, Ängelholm, Sweden) and haematocrit and plasma osmolality was measured using freezing-point depression (Model 3300 Micro-Osmometer; Advanced Instruments Inc., Norwood, MA, USA). Urine and fluid-intake corrected mass changes were calculated between resting and post-exercise masses. Rates of perceived exertion (RPE; Borg 1973) and abdominal discomfort (modified from; Rowlands et al. 2008) were determined as averages over each 15-min interval. Heart rate (HR) was continuously recorded (Polar RS400; Polar Electro, Kempele, Finland) throughout each trial.

Plasma insulin (Insulin ELISA; IBL International GmbH, Hamburg, Germany: Intra-assay CV = 1.8–2.6%), Interleukin-6 (IL-6; Interleukin-6 ELISA; IBL International GmbH, Hamburg, Germany: Intra-assay CV = 0.2–7.8%) and epinephrine (IBL Adrenalin; IBL International GmbH, Hamburg, Germany: Intra-assay CV = 6.8%) concentrations were obtained by enzyme-linked immunosorbent assays (ELISA) techniques. Plasma epinephrine concentrations represent only the following: Pre, 45-min, HT, 60-, 90- and 120-min time-points. Plasma glycerol and non-esterified fatty acid (NEFA) concentrations were measured using a clinical chemistry analyser (RX Daytona; Randox Laboratories Ltd., Co. Antrim, UK: NEFA intra-assay CV = 4.7–4.8%, Glycerol intra-assay CV = 0.9–1.3%).

#### Nutritional interventions

Participants consumed 8% drinks containing maltodextrin (MDX; GI: 90–100), or isomaltulose (PALATINOSE™, PSE; GI: 32) carbohydrate sources (Atkinson et al. 2008) or a fluid-electrolyte placebo (void of carbohydrate but containing an artificial sweetener; PLA) in a double-blind, randomised and cross-over fashion separated by 10 ± 4 days. Trial randomisation was carried out using a Latin-square design (http://www.randomization.com). Drinks were prepared and served in black opaque bottles by an independent person who was not involved in the main trial testing.

Professionally formulated beverage powders (Beneo GmbH, Obrigheim, Germany) were mixed with the same fluid-electrolyte beverage (mineral water; Highland Spring; Highland Spring Group, Perthshire, Scotland) and were matched for taste (orange flavour), texture, colour and sweetness. All participants consumed mineral water with the pre-exercise meal (500 ml) and before commencing the extra-time period of simulated match-play (4.5 ml kg^−1^ body mass). Treatment beverages were ingested during the soccer skill component of the warm-up (4.5 ml kg^−1^ body mass, corresponding to 0.36 g kg^−1^ body mass carbohydrates for the PSE and MDX trials) and at HT (6 ml kg^−1^ body mass, corresponding to 0.48 g kg^−1^ body mass carbohydrates for the PSE and MDX trials). No food or beverages other than that mentioned were consumed throughout testing. Participants were unable to distinguish between trials.

### Statistical analyses

Statistical analyses were carried out using SPSS software (Version 21.0; SPSS Inc., IL, USA). All results are reported as the mean ± standard deviation (SD). The level of statistical significance was set at *P* ≤ 0.05. Data were sampled for normality and two-way repeated measures analyses of variance (ANOVA) established significant effects in the physiological and performance responses attributable to time and/or condition. Mauchly’s test was consulted and Greenhouse–Geisser correction applied if the assumption of sphericity was violated. If a significant time × trial interaction existed, trial was deemed to have influenced the findings and simple main effects were examined using one-way repeated measures ANOVA. LSD corrected post-hoc tests isolated significant findings between trials.

## Results

Pre-trial energy intake (13.8 ± 5.1 MJ day^−1^), energy from individual macronutrients (carbohydrates: 45 ± 7%, fats: 34 ± 7%, proteins: 21 ± 5%) and resting plasma osmolality (283 ± 26 mOsmol kg^−1^) were similar between trials (all *P* > 0.05).

### Physiological responses

**Fig. 2:**
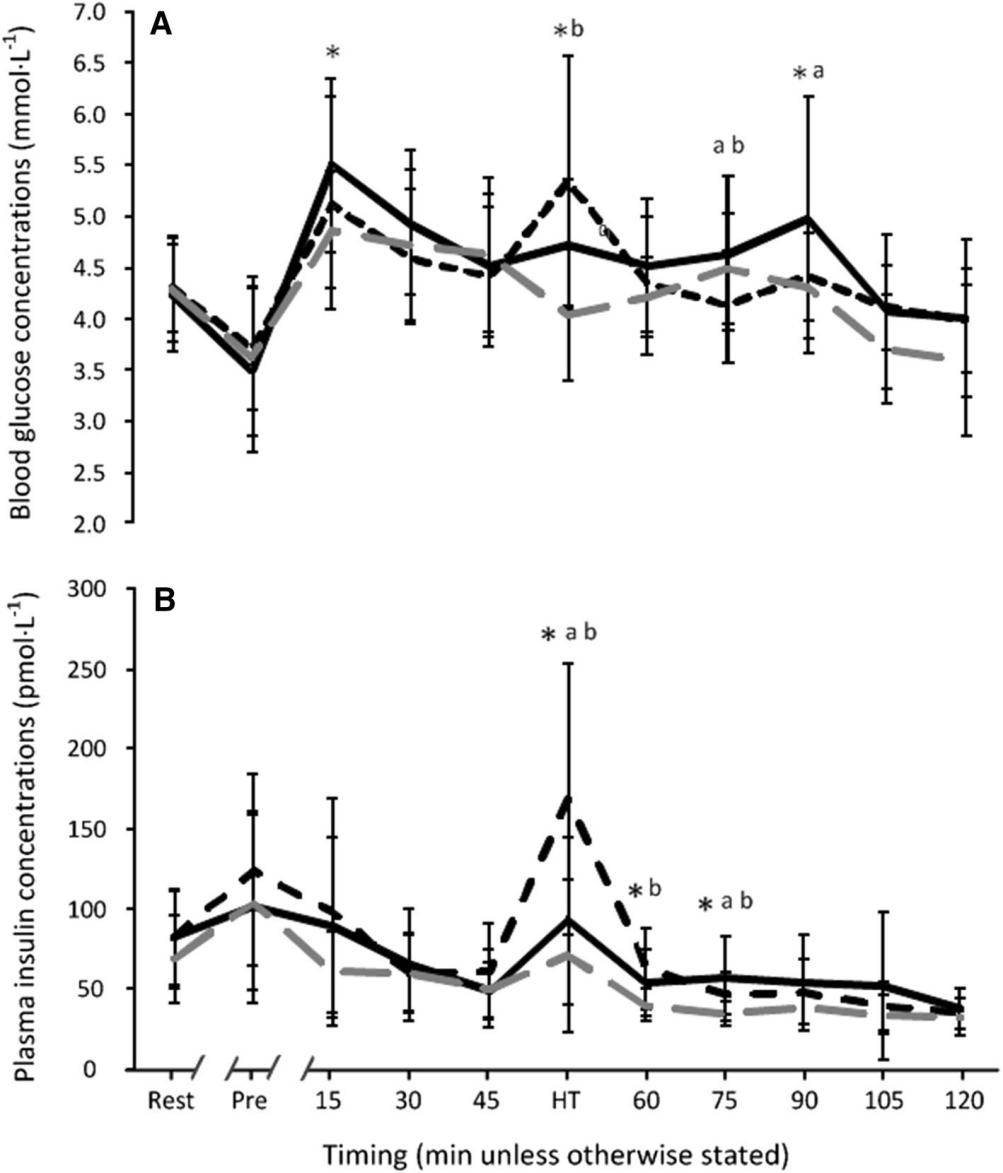
Blood glucose (**a**) and plasma insulin (**b**) concentrations throughout the PSE (black solid line), MDX (black dashed line) and PLA (grey dashed line) trials (*n* = 22, data are presented as mean ± SD). *Significant difference between PSE and PLA at corresponding time-point; **a** represents significant difference between PSE and MDX at corresponding time-point; **b** represents significant difference between MDX and PLA at corresponding time-point

Blood glucose concentrations were influenced by drink (time × trial interaction: *F*_(10,208)_ = 4.577, *P* < 0.001, partial-eta^2^ = 0.179; Fig. 2a). Despite similarities between trials before exercise, blood glucose was higher in PSE (*P* = 0.007), but not MDX (*P* = 0.168), at 15 min when compared to PLA. At HT, blood glucose concentrations in PSE (*P* = 0.001) and MDX (*P* < 0.001) were greater than PLA with a trend for a higher blood glucose peak in MDX compared to PSE (*P* = 0.058). The post HT decline in glycaemia following the onset of the second half was significantly greater with MDX (−19%; −1.0 ± 1.31 mmol l^−1^) compared to PSE (−4%; −0.2 ± 0.75 mmol l^−1^; *P* = 0.015). Blood glucose concentrations at 60 min were similar (*P* = 0.239). At 75 and 90 min, blood glucose was greatest in PSE versus MDX (*P* = 0.010 and *P* = 0.044, respectively) with MDX values at 75 min also being lower than PLA (*P* = 0.038). No further differences were observed across trials.

Blood lactate concentrations were significantly influenced by exercise (time effect: *F*_(2,47)_ = 97.196, *P* < 0.001, partial-eta^2^ = 0.822; Table 1) with values from 15 min being elevated over Pre (all *P* < 0.001) but reducing throughout exercise. Values from 45 min were lower than the start of exercise (all *P* < 0.01). No trial effects existed (time × trial interaction: *F*_(8,161)_ = 0.576, *P* = 0.790, partial-eta^2^ = 0.027).

**Table 1:** Lactate, glycerol, NEFA, IL-6, plasma osmolality and epinephrine responses (*n* = 22, data are presented as means ± SD)

Variable	Trial	Timing										
		Rest	Pre	15-min	30-min	45-min	HT	60-min	75-min	90-min	105-min	120-min
Lactate (mmol l^−1^)	PSE	0.83 ± 0.35	0.80 ± 0.18	5.59 ± 2.17	5.13 ± 2.03	5.02 ± 2.08	2.16 ± 0.94	4.16 ± 2.11	3.89 ± 1.86	4.05 ± 2.01	2.92 ± 1.14	3.15 ± 1.42
	MDX	0.77 ± 0.23	0.91 ± 0.28	5.52 ± 2.03	5.51 ± 1.86	5.26 ± 1.96	2.42 ± 1.20	4.56 ± 1.76	4.19 ± 1.51	4.66 ± 1.99	3.29 ± 1.49	3.67 ± 1.62
	PLA	0.79 ± 0.26	0.85 ± 0.21	5.42 ± 2.41	5.44 ± 2.33	4.94 ± 2.23	2.17 ± 1.06	4.39 ± 1.87	3.97 ± 1.70	4.10 ± 2.13	3.25 ± 1.60	3.73 ± 1.73
Glycerol (mmol l^−1^)	PSE	0.03 ± 0.01	0.02 ± 0.02	0.06 ± 0.02	0.07 ± 0.03	0.09 ± 0.04*	0.07 ± 0.03	0.12 ± 0.06*	0.14 ± 0.04*	0.17 ± 0.05*	0.19 ± 0.07*	0.27 ± 0.09*
	MDX	0.03 ± 0.01	0.02 ± 0.01	0.07 ± 0.04	0.08 ± 0.04	0.09 ± 0.04*	0.08 ± 0.03	0.09 ± 0.04*	0.14 ± 0.05*	0.16 ± 0.07*	0.23 ± 0.08*	0.30 ± 0.07*
	PLA	0.03 ± 0.03	0.02 ± 0.01	0.08 ± 0.03	0.09 ± 0.03	0.11 ± 0.05	0.09 ± 0.03	0.15 ± 0.04	0.19 ± 0.07	0.26 ± 0.06	0.29 ± 0.08	0.36 ± 0.09
NEFA (mmol l^−1^)	PSE	0.23 ± 0.12*	0.06 ± 0.03*^a^	0.17 ± 0.11*	0.18 ± 0.17*	0.30 ± 0.26*	0.64 ± 0.38	0.49 ± 0.37*	0.58 ± 0.32	0.75 ± 0.44*	0.95 ± 0.47*	1.22 ± 0.47*
	MDX	0.32 ± 0.19	0.09 ± 0.04*	0.16 ± 0.13*	0.18 ± 0.11*	0.26 ± 0.16*	0.71 ± 0.41	0.31 ± 0.19*	0.54 ± 0.28*	0.59 ± 0.25*	0.95 ± 0.37*	1.34 ± 0.38
	PLA	0.46 ± 0.30	0.10 ± 0.07	0.25 ± 0.15	0.27 ± 0.18	0.44 ± 0.25	0.81 ± 0.38	0.70 ± 0.30	0.80 ± 0.41	1.03 ± 0.38	1.24 ± 0.40	1.50 ± 0.42
IL-6 (pg ml^−1^)	PSE	3.66 ± 1.93	3.52 ± 2.31	4.10 ± 2.06	4.15 ± 2.17	4.85 ± 2.57	5.19 ± 1.97	5.41 ± 2.58	6.07 ± 2.90	8.18 ± 3.47	9.57 ± 3.95	11.47 ± 5.54
	MDX	4.32 ± 2.50	4.63 ± 2.57	4.79 ± 2.47	5.85 ± 2.56	6.34 ± 2.66	6.81 ± 3.61	7.10 ± 2.99	7.61 ± 3.40	8.87 ± 3.90	11.63 ± 4.67	14.99 ± 9.52
	PLA	3.74 ± 2.32	4.49 ± 2.41	5.36 ± 3.85	5.43 ± 2.33	7.38 ± 5.95	7.23 ± 3.42	6.85 ± 3.25	7.09 ± 2.34	9.56 ± 3.85	10.16 ± 3.72	14.32 ± 5.74
Osmolality (mOsmol kg^−1^)	PSE	274 ± 41	283 ± 33	279 ± 38^a^	290 ± 37	292 ± 38	276 ± 33	292 ± 47	297 ± 16	296 ± 13	300 ± 11	294 ± 6^a^
	MDX	284 ± 13	296 ± 16	304 ± 15*	296 ± 16	287 ± 26	290 ± 10	292 ± 12	300 ± 21	288 ± 21	299 ± 17	285 ± 9*
	PLA	292 ± 12	298 ± 12	293 ± 23	295 ± 17	293 ± 14	290 ± 13	294 ± 9	297 ± 9	299 ± 10	292 ± 10	296 ± 17
Epinephrine (pmol l^−1^)	PSE	–	255.6 ± 89.0	–	–	464.5 ± 257.0	274.6 ± 116.0	589.8 ± 311.4	–	586.1 ± 349.6*^a^	–	996.3 ± 611.9*
	MDX	–	285.3 ± 80.1	–	–	470.8 ± 205.9	326.3 ± 106.9	681.2 ± 561.4	–	877.8 ± 484.5	–	1397.2 ± 775.2*
	PLA	–	253.6 ± 84.3	–	–	695.4 ± 555.0	288.6 ± 82.5	627.9 ± 217.7	–	1313.7 ± 975.4	–	3149.5 ± 2265.1

*Significant difference compared to PLA at corresponding time-point

^a^Significant difference compared to MDX at corresponding time-point

Plasma insulin was similar between conditions until HT (time × trial interaction: *F*_(6,130)_ = 5.894, *P* < 0.001, partial-eta^2^ = 0.219; Fig. 2b) where PSE (*P* = 0.031) and MDX (*P* < 0.001) demonstrated higher values compared to PLA; the rise in insulin was greatest in MDX (*P* = 0.002 versus PSE). At 60 and 75 min, insulin concentrations remained elevated over PLA in MDX (*P* = 0.001 and *P* = 0.001, respectively) and PSE (*P* = 0.004 and *P* < 0.001, respectively). Differences between MDX and PSE at 75 min (*P* = 0.040) were negated at 90 min, with no differences observed between carbohydrate trials at the end of exercise.

Plasma epinephrine was influenced by trial and exercise (time × trial interaction: *F*_(2,42)_ = 12.342, *P* < 0.001, partial-eta^2^ = 0.370; Table 1) with similar concentrations between conditions until 90 min where PSE (*P* = 0.004), but not MDX (*P* = 0.108), attenuated the rise seen in PLA. Epinephrine values were lower in PSE compared to MDX at 90 min (*P* = 0.030). At 120 min, both MDX (*P* = 0.002) and PSE (*P* < 0.001) attenuated the rise observed in PLA with a trend for lower epinephrine concentrations with PSE compared to MDX (*P* = 0.073).

Glycerol was influenced by time and trial (interaction: *F*_(7,141)_ = 9.025, *P* < 0.001, partial-eta^2^ = 0.301; Table 1). No between-trial differences existed until 45 min where MDX (*P* = 0.017) and PSE (*P* = 0.028) attenuated the rise observed in PLA. At HT, glycerol concentrations were similar between conditions but from 60 min onwards both MDX (all *P* < 0.05) and PSE (all *P* < 0.05) attenuated the rise in glycerol seen in PLA with no difference between carbohydrate trials (all *P* > 0.05). Similarly, NEFA concentrations were influenced by drink (time × trial interaction: *F*_(7,149)_ = 2.517, *P* = 0.017, partial-eta^2^ = 0.107; Table 1) with greater values observed in PLA at all time-points except for HT.

Exercise influenced the IL-6 response (time effect: *F*_(4,73)_ = 82.708, *P* < 0.001, partial-eta^2^ = 0.798) with significant increases from resting values observed at all time-points (all *P* ≤ 0.001). Further increases in IL-6 were observed during extra time (i.e., at 105 and 120 min) relative to 90 min (both *P* ≤ 0.001). No interaction between time and condition was present (time × trial interaction: *F*_(4,93)_ = 0.979, *P* = 0.429, partial-eta^2^ = 0.045; Table 1). However, there was a trend for IL-6 concentrations being overall reduced (trial effect: *F*_(2,33)_ = 3.324, *P* = 0.060, partial-eta^2^ = 0.137) with PSE compared to MDX (*P* = 0.014) and PLA (*P* = 0.020).

Plasma osmolality varied according to trial (time × trial interaction: *F*_(5,110)_ = 2.611, *P* = 0.026, partial-eta^2^ = 0.111; Table 1) with values at 15 min being lower in PSE when compared to MDX (*P* = 0.014). Notably, osmolality in PLA was also lower than MDX (*P* = 0.034) at 15 min. No further differences were observed until 120 min (MDX versus PSE: *P* < 0.001, MDX versus PLA: *P* = 0.006). Body mass reduced by 1.82 ± 0.97, 1.63 ± 0.48 and 1.82 ± 0.67 kg throughout PSE, MDX and PLA, respectively, but the magnitude of change was similar between trials (time × trial interaction: *F*_(1,30)_ = 0.806, *P* = 0.411, partial-eta^2^ = 0.037).

Abdominal discomfort varied throughout exercise according to trial (time × trial interaction: *F*_(7,139)_ = 2.418, *P* = 0.025, partial-eta^2^ = 0.103; Table 2). Although post-hoc tests were unable to locate these differences, ratings of abdominal discomfort were consistently lower with both carbohydrates compared to PLA from 60 min onwards. Exercise influenced abdominal discomfort (time effect: *F*_(2,38)_ = 32.736, *P* < 0.001, partial-eta^2^ = 0.609) with significant increases throughout exercise from 15 min onwards (all *P* < 0.001) and during extra-time (i.e., 105–120 min versus 75–90 min: *P* < 0.001).

**Table 2:** Peak HR, mean HR, RPE and abdominal discomfort responses (*n* = 22, data are presented as means ± SD)

Variable	Trial	Timing							
		0–15 min	15–30 min	30–45 min	45–60 min	60–75 min	75–90 min	90–105 min	105–120 min
Peak HR (beats min^−1^)	PSE	184 ± 9	184 ± 9	183 ± 8	184 ± 7	188 ± 13	188 ± 13	184 ± 8	189 ± 12
	MDX	188 ± 10	187 ± 8	186 ± 8	186 ± 9	188 ± 9	187 ± 9	186 ± 10	189 ± 9
	PLA	184 ± 11	183 ± 11	184 ± 10	183 ± 10	185 ± 10	185 ± 9	184 ± 9	187 ± 10
Mean HR (beats min^−1^)	PSE	159 ± 9	158 ± 11	159 ± 10	160 ± 7	158 ± 10	160 ± 11	159 ± 9	161 ± 10
	MDX	164 ± 11	162 ± 11	163 ± 10	161 ± 11	163 ± 11	162 ± 11	164 ± 10	163 ± 10
	PLA	161 ± 13	159 ± 15	159 ± 16	160 ± 11	161 ± 14	161 ± 12	162 ± 12	164 ± 13
RPE (units)	PSE	11.6 ± 1.9	12.8 ± 1.6	13.4 ± 1.3	13.2 ± 1.4	14.5 ± 1.1	15.1 ± 1.4	15.4 ± 1.6	16.7 ± 1.9
	MDX	11.3 ± 1.8	12.6 ± 1.4	13.3 ± 1.4	12.7 ± 1.6	14.1 ± 1.6	14.8 ± 1.6	15.2 ± 2.1	16.6 ± 2.1
	PLA	11.6 ± 2.1	12.9 ± 2.0	13.5 ± 1.8	13.2 ± 1.9	14.4 ± 2.0	15.1 ± 1.9	15.5 ± 1.9	16.8 ± 2.0
Abdominal discomfort (units)	PSE	1.6 ± 1.4	2.3 ± 1.8	2.4 ± 1.6	2.3 ± 1.5	2.5 ± 1.7	2.9 ± 1.8	3.2 ± 1.8	3.7 ± 2.0
	MDX	1.6 ± 0.9	2.2 ± 1.3	2.5 ± 1.4	2.3 ± 1.5	2.5 ± 1.5	2.9 ± 1.7	3.1 ± 1.8	4.0 ± 2.2
	PLA	1.6 ± 1.2	2.2 ± 1.4	2.5 ± 1.5	2.4 ± 1.5	3.0 ± 1.6	3.5 ± 1.8	3.6 ± 1.9	4.4 ± 2.3

Exercise (time effect: *F*_(2,33)_ = 86.330, *P* < 0.001, partial-eta^2^ = 0.804), but not trial (time × trial interaction: *F*_(5,98)_ = 0.326, *P* = 0.886, partial-eta^2^ = 0.015; Table 2), influenced RPE after 0–15 min (all *P* < 0.05). Further increases were observed in extra-time relative to 75–90 min (*P* < 0.001). Mean and peak HR were comparable between conditions (time × trial interaction: *F*_(6,133)_ = 0.995, *P* = 0.434, partial-eta^2^ = 0.045; time × trial interaction: *F*_(4,88)_ = 1.056, *P* = 0.385, partial-eta^2^ = 0.048, respectively; Table 2) with greater values observed at the end of exercise compared to the first 15 min (mean HR: *P* = 0.022; peak HR: *P* = 0.012).

### Physical performance

Exercise reduced 20-m repeated sprint performance (time effect: *F*_(4,84)_ = 8.234, *P* < 0.001, partial-eta^2^ = 0.282) with sprint decrement values from Pre 2nd half onwards being greater than Pre 1st half (all *P* < 0.05). Repeated sprint performance reduced similarly throughout exercise between trials (time × trial interaction: *F*_(4,87)_ = 1.552, *P* = 0.193, partial-eta^2^ = 0.069; Table 3). Exercise (time effect: *F*_(3,53)_ = 9.087, *P* < 0.001, partial-eta^2^ = 0.302), but not trial (time × trial interaction: *F*_(5,100)_ = 1.098, *P* = 0.365, partial-eta^2^ = 0.050; Table 3), influenced jump height. A transient reduction in jump height compared to Pre 1st half occurred after HT (i.e., at Pre 2nd half; *P* = 0.001). Jump performance reduced further after the extra-time period with jump height being lower than Pre 1st half values (*P* = 0.029) and also lower compared to Post 2nd half (*P* = 0.024). Mean 15 m sprint velocity reduced throughout exercise (time effect: *F*_(3,66)_ = 96.872, *P* < 0.001, partial-eta^2^ = 0.822), but no effect of drink was observed (time × trial interaction: *F*_(5,113)_ = 0.754, *P* = 0.594, partial-eta^2^ = 0.035; Table 4). All sprints performed from 15–30 min onwards were slower than 0–15 min values (all *P* < 0.05) and further decrements were observed during the extra-time period relative to 75–90 min (*P* < 0.001).

**Table 3:** Peak 20-m sprint velocity, sprint decrement index, jump height, shot speed, shot precision and shot success responses (*n* = 22, data are presented as means ± SD)

Variable	Trial	Timing				
		Pre 1st half	Post 1st half	Pre 2nd half	Post 2nd half	Post extra-time
Peak 20-m sprint velocity (m s^−1^)	PSE	6.27 ± 0.27	6.15 ± 0.33	5.98 ± 0.32	5.98 ± 0.38	5.82 ± 0.51
	MDX	6.24 ± 0.27	6.12 ± 0.31	5.98 ± 0.27	5.97 ± 0.38	5.77 ± 0.53
	PLA	6.26 ± 0.29	6.14 ± 0.29	6.00 ± 0.29	6.11 ± 0.34	5.84 ± 0.47
Sprint decrement index (%)	PSE	3.5 ± 2.1	3.0 ± 1.4	4.8 ± 4.0	5.1 ± 2.8	5.8 ± 4.1
	MDX	3.5 ± 2.2	5.1 ± 3.5	4.6 ± 2.8	3.9 ± 2.5	5.6 ± 3.8
	PLA	3.8 ± 2.1	3.9 ± 2.7	4.1 ± 3.0	6.4 ± 4.0	6.5 ± 5.2
Jump height (cm)	PSE	34.5 ± 4.4	34.0 ± 4.4	32.6 ± 5.0	33.2 ± 4.3	32.1 ± 5.3
	MDX	33.9 ± 4.8	34.1 ± 5.7	32.3 ± 5.3	33.7 ± 5.8	33.1 ± 6.0
	PLA	33.7 ± 4.8	34.7 ± 5.3	32.6 ± 5.6	33.9 ± 6.1	33.2 ± 6.1
Shot speed (m s^−1^)	PSE	18.9 ± 2.0	18.7 ± 2.0	18.0 ± 2.1	18.5 ± 1.9	18.0 ± 1.9
	MDX	18.2 ± 2.2	17.9 ± 1.9	18.2 ± 1.7	17.6 ± 1.6	17.8 ± 2.0
	PLA	18.7 ± 2.3	18.3 ± 2.4	17.9 ± 2.5	18.4 ± 2.3	17.6 ± 2.5
Shot precision (cm)	PSE	132 ± 54	138 ± 70	146 ± 67	121 ± 36	156 ± 64
	MDX	139 ± 53	118 ± 62	153 ± 74	121 ± 54	128 ± 53
	PLA	124 ± 52	116 ± 51	137 ± 64	143 ± 52	120 ± 63
Shot success (%)	PSE	67 ± 25	65 ± 21	76 ± 20	78 ± 21	69 ± 24
	MDX	66 ± 21	67 ± 20	64 ± 29	72 ± 22	74 ± 24
	PLA	67 ± 22	69 ± 20	74 ± 25	75 ± 20	66 ± 20

**Table 4:** Mean 15-m sprint velocity, dribbling speed, dribbling precision and dribbling success responses (*n* = 22, data are presented as means ± SD)

Variable	Trial	Timing							
		0–15 min	15–30 min	30–45 min	45–60 min	60–75 min	75–90 min	90–105 min	105–120 min
Mean 15-m sprint velocity (m s^−1^)	PSE	5.58 ± 0.36	5.48 ± 0.35	5.48 ± 0.34	5.40 ± 0.35	5.28 ± 0.38	5.19 ± 0.41	5.09 ± 0.42	4.97 ± 0.50
	MDX	5.54 ± 0.37	5.43 ± 0.39	5.37 ± 0.41	5.29 ± 0.32	5.21 ± 0.35	5.15 ± 0.37	5.00 ± 0.40	4.84 ± 0.48
	PLA	5.62 ± 0.38	5.56 ± 0.43	5.45 ± 0.41	5.44 ± 0.39	5.26 ± 0.31	5.16 ± 0.33	5.06 ± 0.30	4.89 ± 0.32
Dribbling speed (m s^−1^)	PSE	3.2 ± 0.3	3.2 ± 0.2	3.2 ± 0.4	3.2 ± 0.2	3.3 ± 0.3	3.2 ± 0.4	3.3 ± 0.3	3.0 ± 0.3
	MDX	3.3 ± 0.4	3.2 ± 0.2	3.2 ± 0.3	3.3 ± 0.4	3.3 ± 0.4	3.2 ± 0.4	3.2 ± 0.3	3.1 ± 0.3
	PLA	3.2 ± 0.3	3.2 ± 0.2	3.1 ± 0.2	3.2 ± 0.2	3.2 ± 0.2	3.2 ± 0.3	3.1 ± 0.2	3.1 ± 0.3
Dribbling precision (cm)	PSE	42 ± 13	41 ± 10	40 ± 11	40 ± 10	35 ± 9	35 ± 8	37 ± 6	37 ± 11
	MDX	44 ± 8	40 ± 8	41 ± 8	38 ± 8	36 ± 10	35 ± 10	37 ± 9	36 ± 9
	PLA	41 ± 8	37 ± 8	37 ± 8	34 ± 7	33 ± 8	35 ± 9	34 ± 8	34 ± 7
Dribbling success (%)	PSE	91 ± 9	80 ± 15	92 ± 11	93 ± 6	88 ± 11	86 ± 12	89 ± 8	79 ± 14
	MDX	90 ± 8	90 ± 13	90 ± 13	94 ± 8	93 ± 8	90 ± 13	90 ± 11	87 ± 10
	PLA	90 ± 8	89 ± 8	91 ± 11	93 ± 8	86 ± 15	86 ± 13	88 ± 11	90 ± 8

### Skilled performance

Shot precision (time effect: *F*_(3,61)_ = 1.256, *P* = 0.297, partial-eta^2^ = 0.056) and success (time effect: *F*_(4,84)_ = 1.350, *P* = 0.258, partial-eta^2^ = 0.060) were unaffected by exercise, whereas there was a trend for shot speed being reduced over the trial duration (time effect: *F*_(2,40)_ = 3.174, *P* = 0.055, partial-eta^2^ = 0.131). Ball speed was 2.9% slower at Pre 2nd half when compared to shots taken before exercise (*P* = 0.039). Shot speeds Post extra-time were 4.3% (*P* = 0.049) slower than Pre values and 2.9% (*P* = 0.039) slower than Post 2nd half. Shot success was slightly higher with PSE compared to MDX at Pre and Post 2nd half (76 versus 64% and 78 versus 72%, respectively; Table 3). After 90 min (i.e., at Post 2nd half), more players scored 100% of shots when they had taken PSE (41%) rather than MDX (23%) or PLA (23%). However, the effects of drinks on soccer shooting performance throughout exercise were non-significant (speed: time × trial interaction: *F*_(8,168)_ = 1.381, *P* = 0.208, partial-eta^2^ = 0.062; precision: time × trial interaction: *F*_(8,168)_ = 1.753, *P* = 0.090, partial-eta^2^ = 0.077; success: time × trial interaction: *F*_(8,168)_ = 0.891, *P* = 0.525, partial-eta^2^ = 0.041; Table 3).

Exercise reduced dribble speed (time effect: *F*_(7,147)_ = 5.774, *P* < 0.001, partial-eta^2^ = 0.216), with dribbles performed at 30–45 min (*P* = 0.008) and throughout extra-time (i.e., at 90–105-min; *P* = 0.067, and 105–120 min; *P* < 0.001), being slower when compared to 0–15 min. Dribble speed reduced further during extra-time (i.e., 105–120-min) when compared to 75–90 min (*P* = 0.002). Dribbling precision improved throughout exercise (time effect: *F*_(4,92)_ = 16.777, *P* < 0.001, partial-eta^2^ = 0.444) with dribbles performed from 30–45 min onwards being closer to the target when compared to 0–15 min (all *P* ≤ 0.029). Dribble success reduced throughout exercise (time effect: *F*_(7,147)_ = 6.380, *P* < 0.001, partial-eta^2^ = 0.233) as dribbles were 5.2% less successful at the end of extra-time versus 0–15 min (*P* < 0.001). Dribbling performances throughout exercise were comparable between beverages (time × trial interaction for speed: *F*_(7,137)_ = 0.886, *P* = 0.514, partial-eta^2^ = 0.041, time × trial interaction for precision: *F*_(6,120)_ = 0.662, *P* = 0.674, partial-eta^2^ = 0.031; Table 4), yet an interaction existed for success (time × trial interaction *F*_(7,138)_ = 2.133, *P* = 0.048, partial-eta^2^ = 0.092; Table 4).

## Discussion

Although carbohydrates did not attenuate performance declines throughout prolonged simulated soccer match-play, physiological responses differed according to the ingestion of either ~ 20 g h^−1^ of the low-GI carbohydrate isomaltulose or high-GI maltodextrin. When compared to MDX, and while eliciting similar abdominal discomfort values, blood glucose concentrations were greater between 75 and 90 min in PSE and the magnitude of a transient decline in glycaemia observed at the start of the second half (i.e., 60 min) was lessened. Moreover, PSE was measurably more effective than MDX in lowering the epinephrine response to prolonged exercise. Thus, when limited opportunities exist to supply exogenous energy during prolonged intermittent exercise, low-GI carbohydrates may offer an alternative strategy to high-GI carbohydrate consumption.

In an attempt to maintain glycaemia and to enhance exogenous carbohydrate oxidation (Coyle et al. 1986) team sports athletes are recommended to consume carbohydrate–electrolyte beverages before and during exercise (Convertino et al. 1996; Thomas et al. 2016). Paradoxically, a transient lowering of blood glucose concentrations occurs in the initial stages of the second half when carbohydrate is consumed frequently (Bangsbo et al. 2007; Kingsley et al. 2014; Krustrup et al. 2006; Russell et al. 2012, 2014) and likely reflects physiological differences between the effects of carbohydrates consumed in either the passive (i.e., HT; insulin secretion attempts to normalise blood glucose concentrations by decreasing lipolysis and facilitating glucose uptake) versus active (i.e., exercise; counter-regulatory hormones dampen the insulin response) states combined with the insulin-independent glucose uptake that occurs after a prior exercise bout. In studies aiming to elicit rebound hypoglycaemia, consuming low-GI carbohydrates reduced the number of participants experiencing blood glucose concentrations < 3.5 mmol l^−1^, a finding attributed to the degree of hyperinsulinemia resulting from pre-exercise carbohydrate ingestion (Costill et al. 1977; Jentjens and Jeukendrup 2003). Indeed, we observed lower HT insulin concentrations in PSE when compared to MDX (Fig. 2b) and fewer participants experienced hypoglycaemia (defined as glucose concentrations < 3.8 mmol l^−1^; Balijepalli et al. 2017; Smith et al. 2016) at 60 min (18 versus 27%). Moreover, the change in glycaemia from HT was also less in PSE compared to MDX (− 4%; − 0.2 ± 0.75 mmol l^−1^ versus − 19%; − 1.0 ± 1.31 mmol l^−1^; *P* = 0.015) at 60 min (Fig. 2a). Although the effects of rapid reductions in glycaemia are unclear, blood glucose concentrations appeared better maintained at the start of the second half of soccer-specific exercise when low-GI carbohydrates were consumed.

Although glycogen measurements were not taken in the present study, it is likely that the additional demands of extra-time further challenged muscle and liver glycogen reserves (Foskett et al. 2008). Metabolites associated with greater net muscle glycogen utilisation such as plasma epinephrine and IL-6 (Febbraio et al. 1998; Helge et al. 2003) which were significantly elevated over 90-min values at 120 min would support this supposition. Likewise, post-exercise NEFA and glycerol responses are indicative of significant taxing of endogenous energy stores. As fatigue during high-intensity exercise has previously been attributed to muscle glycogen depletion (Foskett et al. 2008); our findings further support the additive effects of the soccer extra-time period when compared to the previous 90 min (Harper et al. 2016a). Notably, carbohydrate ingestion has been shown to blunt epinephrine release during prolonged exercise (Felig et al. 1982; Fritzsche et al. 2000), but this response appears minimised during 90 min of exercise when the carbohydrate dose is low (~ 13 g h^−1^; Clarke et al. 2008). Ingestion of ~ 20 g h^−1^ of carbohydrate in the current study, albeit a low but practically applicable dose, yielded reductions in epinephrine concentrations at 90 min in PSE and at 120 min in both PSE and MDX (with epinephrine levels being lower in PSE compared to MDX; *P* = 0.030 and *P* = 0.073 at 90 and 120 min, respectively). The practical implications of this blunted response are unclear as performance was not affected in this study.

In agreement with previous authors, IL-6 concentrations increased with exercise duration and peaked after exercise (Souglis et al. 2015). The prolonged nature of the exercise protocol likely explains the differences in the magnitude of post-exercise IL-6 responses (Table 1) compared to previous authors investigating intermittent exercise (Andersson et al. 2010; Souglis et al. 2015). Due to methodological limitations, the mechanism causing the increase in IL-6 concentrations could not be isolated but exercise intensity and duration (Gokhale et al. 2007), the occurrence of hypoglycaemia (Dotson et al. 2008) and supraphysiological doses of epinephrine (Helge et al. 2003) have been shown to modulate the circulatory appearance of IL-6 or IL-6 mRNA expression. Although likely attributable to the low dose of carbohydrate used (~ 20 g h^−1^), no time × trial interaction effect was found for IL-6 concentrations in the present study. Our data did indicate a trend for overall reduced IL-6 levels with PSE when compared to MDX and PLA, supporting the findings of Kraemer et al. (2015), albeit when isomaltulose was consumed with beta-hydroxy-beta-methylbutyrate in a recovery context. Carbohydrate provision may have important consequences for soccer performance in times of increased exercise volume as reduced glycogen availability, changes in calcium homeostasis and increased formation of reactive oxygen species can activate transcription factors which regulate IL-6 synthesis (Fischer 2006).

Ingesting fluid in a manner representing better ecological validity than examined previously (i.e., at breakfast, during the warm-up and HT and before extra-time), resulted in post-exercise mass losses that exceeded 2% body mass. It, therefore, appears that despite players arriving in a fed and hydrated state and consuming 0.5 L of fluid with breakfast, and ingesting 0.4–0.5 L h^−1^ during exercise, such volumes were not enough to prevent mass losses during intermittent exercise performed in ambient temperatures. However, although post-hoc analyses were unable to isolate specific between-trial differences, abdominal discomfort values (Table 2) were lower for MDX and PSE when compared to PLA from 60 min onwards; findings which are contrary to those observed when substantial amounts of isomaltulose (> 60 g h^−1^) were ingested before and during 2 h of cycling at 60% of maximum workload (Oosthuyse et al. 2015). Accordingly, such findings may have important implications for applied practitioners who may have previously been deterred from recommending low-GI carbohydrates before soccer-specific exercise due to the risk of elevating abdominal discomfort.

Ingesting carbohydrates at a rate of ~ 20 g h^−1^ did not significantly attenuate reductions in physical (i.e., sprinting and jumping) or skilled (i.e., soccer shooting and dribbling) performances observed throughout 120 min of simulated match-play. For soccer skills, a speed–accuracy trade-off occurred as shooting and dribbling speed reduced throughout exercise. These findings are in contrast to those that have demonstrated ergogenic effects as a result of higher amounts of carbohydrate (> 40 g h^−1^) ingested more frequently (Ali and Williams 2009; Russell et al. 2012). Although regular fluid-ingestion does not align with the hydro-nutritional strategies of competing soccer players, it is likely that reduced opportunities to ingest carbohydrate in fluid form likely precluded players from achieving ergogenic thresholds of consumption (Ali and Williams 2009; Russell and Kingsley 2014). Therefore, when limited opportunities exist to consume fluids containing carbohydrates, such strategies may be supplemented by alternative modes of carbohydrate provision such as gels (Harper et al. 2016a) or by using greater concentrations of carbohydrate solution (Harper et al. 2017). Consideration should, however, still be given to the GI of the carbohydrates consumed (Kingsley et al. 2014).

In addition to the performance decrements seen over the 120-min protocol, transient reductions in physical and skilled performance occurred at the start of the second half as indicated by reduced shot speed and jump height. Therefore, strategies which maintain body temperature over the HT period may be efficacious at the onset of the second half (Russell et al. 2015c). Although markers of muscle and core temperature were not recorded in this study, previous findings show that reductions in jump performance, attributable to losses in core temperature (and likely muscle temperature also), occur throughout a simulated HT break (Russell et al. 2015b). Speculatively, body temperature losses may also explain the observed decrements in skilled as well as physical performances at the onset of the second half.

## Conclusions

In conclusion, this study aimed to examine the physiological and performance effects of high- and low-GI carbohydrates consumed at two time-points before and during 120 min of simulated soccer-specific exercise. Although provision of ~ 20 g h^−1^ of carbohydrate, from either high- or low-GI sources did not attenuate reductions in markers of physical or skill performance, blood glucose concentrations were better maintained throughout exercise and the magnitude of the exercise-induced rebound glycaemic response was lowered in PSE when compared to MDX. Furthermore, isomaltulose proved more effective in lowering the epinephrine response to prolonged exercise. Therefore, when limited opportunities exist to consume fluids during exercise, the consumption of low-GI isomaltulose before soccer-specific exercise may offer an alternative to the use of high-GI carbohydrates (e.g. maltodextrin). Notably, such responses were observed in players who commenced exercise in a fed and hydrated state, and who consumed treatment beverages in a manner that reflects the competitive practices of professional athletes (i.e., ingestion before exercise and at HT). Thus, the match-day practices of soccer players may be modified to include low-GI carbohydrate consumption.

## Abbreviations

ANOVA: Analysis of variance
CV: Coefficient of variation
ELISA: Enzyme-linked immunosorbent assay
GI: Glycaemic index
HR: Heart rate
HT: Half time
IL6: Interleuken-6
LSD: Least significant difference
MDX: Maltodextrin
MJ: Megajoules
NEFA: Non-esterified fatty acids
PLA: Placebo
PSE: Palatinose
PT: Performance test
RPE: Rate of perceived exertion
SD: Standard deviation
SMS: Soccer match simulation

## References

[CR1] Ali A, Williams C (2009). Carbohydrate ingestion and soccer skill performance during prolonged intermittent exercise. J Sports Sci.

[CR2] Andersson H, Bohn SK, Raastad T, Paulsen G, Blomhoff R, Kadi F (2010). Differences in the inflammatory plasma cytokine response following two elite female soccer games separated by a 72-h recovery. Scand J Med Sci Sports.

[CR3] Atkinson FS, Foster-Powell K, Brand-Miller JC (2008). International tables of glycemic index and glycemic load values: 2008. Diabetes Care.

[CR4] Balijepalli C, Druyts E, Siliman G, Joffres M, Thorlund K, Mills EJ (2017). Hypoglycemia: a review of definitions used in clinical trials evaluating antihyperglycemic drugs for diabetes. Clin Epidemiol.

[CR5] Bangsbo J, Iaia FM, Krustrup P (2007). Metabolic response and fatigue in soccer. Int J Sports Physiol Perform.

[CR6] Borg GAV (1973). Perceived exertion—note on history and methods. Med Sci Sports Exerc.

[CR7] Chryssanthopoulos C, Hennessy LC, Williams C (1994). The influence of pre-exercise glucose ingestion on endurance running capacity. Br J Sports Med.

[CR8] Clarke ND, Drust B, Maclaren DP, Reilly T (2008). Fluid provision and metabolic responses to soccer-specific exercise. Eur J Appl Physiol.

[CR9] Convertino VA, Armstrong LE, Coyle EF, Mack GW, Sawka MN, Senay LC, Sherman WM (1996). American College of Sports Medicine position stand—exercise and fluid replacement. Med Sci Sports Exerc.

[CR10] Costill DL, Coyle E, Dalsky G, Evans W, Fink W, Hoopes D (1977). Effects of elevated plasma FFA and insulin on muscle glycogen usage during exercise. J Appl Physiol Respir Environ Exerc Physiol.

[CR11] Coyle EF, Coggan AR, Hemmert MK, Ivy JL (1986). Muscle glycogen utilization during prolonged strenuous exercise when fed carbohydrate. J Appl Physiol.

[CR12] Dahlqvist A, Auricchio S, Semenza G, Prader A (1963). Human intestinal disaccharidases and hereditary disaccharide intolerance. The hydrolysis of sucrose, isomaltose, palatinose (isomaltulose), and a 1,6-alpha-oligosaccharide (isomalto-oligosaccharide) preparation. J Clin Invest.

[CR13] Dotson S, Freeman R, Failing HJ, Adler GK (2008). Hypoglycemia increases serum interleukin-6 levels in healthy men and women. Diabetes Care.

[CR14] Febbraio MA, Lambert DL, Starkie RL, Proietto J, Hargreaves M (1998). Effect of epinephrine on muscle glycogenolysis during exercise in trained men. J Appl Physiol.

[CR15] Felig P, Cherif A, Minagawa A, Wahren J (1982). Hypoglycemia during prolonged exercise in normal men. N Engl J Med.

[CR16] Fischer CP (2006). Interleukin-6 in acute exercise and training: what is the biological relevance?. Exerc Immunol Rev.

[CR17] Foskett A, Williams C, Boobis L, Tsintzas K (2008). Carbohydrate availability and muscle energy metabolism during intermittent running. Med Sci Sports Exerc.

[CR18] Fritzsche RG, Switzer TW, Hodgkinson BJ, Lee SH, Martin JC, Coyle EF (2000). Water and carbohydrate ingestion during prolonged exercise increase maximal neuromuscular power. J Appl Physiol.

[CR19] Gokhale R, Chandrashekara S, Vasanthakumar KC (2007). Cytokine response to strenuous exercise in athletes and non-athletes-an adaptive response. Cytokine.

[CR20] Harper LD, West DJ, Stevenson E, Russell M (2014). Technical performance reduces during the extra-time period of professional soccer match-play. PLoS One.

[CR21] Harper LD, Briggs MA, McNamee G, West DJ, Kilduff LP, Stevenson E, Russell M (2016). Physiological and performance effects of carbohydrate gels consumed prior to the extra-time period of prolonged simulated soccer match-play. J Sci Med Sport.

[CR22] Harper LD, Hunter R, Parker P (2016). Test–retest reliability of physiological and performance responses to 120 min of simulated soccer match-play. J Strength Cond Res.

[CR23] Harper LD, Stevenson EJ, Rollo I, Russell M (2017) The influence of a 12% carbohydrate-electrolyte beverage on self-paced soccer-specific exercise performance. J Sci Med Sport 10.1016/j.jsams.2017.04.015 28483560

[CR24] Helge JW, Stallknecht B, Pedersen BK, Galbo H, Kiens B, Richter EA (2003). The effect of graded exercise on IL-6 release and glucose uptake in human skeletal muscle. J Physiol.

[CR25] Jentjens RL, Jeukendrup AE (2003). Effects of pre-exercise ingestion of trehalose, galactose and glucose on subsequent metabolism and cycling performance. Eur J Appl Physiol.

[CR26] Kingsley M, Penas-Ruiz C, Terry C, Russell M (2014). Effects of carbohydrate-hydration strategies on glucose metabolism, sprint performance and hydration during a soccer match simulation in recreational players. J Sci Med Sport.

[CR27] Kraemer WJ, Hooper DR, Szivak TK (2015). The addition of beta-hydroxy-beta-methylbutyrate and isomaltulose to whey protein improves recovery from highly demanding resistance exercise. J Am Coll Nutr.

[CR28] Krustrup P, Mohr M, Steensberg A, Bencke J, Kjaer M, Bangsbo J (2006). Muscle and blood metabolites during a soccer game: implications for sprint performance. Med Sci Sports Exerc.

[CR29] MacDonald I, Daniel JW (1983). The bio-availability of isomaltulose in man and rat. Nutr Rep Int.

[CR30] Oosthuyse T, Carstens M, Millen AM (2015). Ingesting isomaltulose versus fructose-maltodextrin during prolonged moderate-heavy exercise increases fat oxidation but impairs gastrointestinal comfort and cycling performance. Int J Sport Nutr Exerc Metab.

[CR31] Ramsbottom R, Brewer J, Williams C (1988). A progressive shuttle run test to estimate maximal oxygen uptake. Br J Sports Med.

[CR32] Rowlands DS, Thorburn MS, Thorp RM, Broadbent S, Shi X (2008). Effect of graded fructose coingestion with maltodextrin on exogenous 14C-fructose and 13C-glucose oxidation efficiency and high-intensity cycling performance. J Appl Physiol.

[CR33] Russell M, Kingsley M (2014). The efficacy of acute nutritional interventions on soccer skill performance. Sports Med.

[CR34] Russell M, Benton D, Kingsley M (2010). Reliability and construct validity of soccer skills tests that measure passing, shooting, and dribbling. J Sports Sci.

[CR35] Russell M, Benton D, Kingsley M (2011). The effects of fatigue on soccer skills performed during a soccer match simulation. Int J Sports Physiol Perform.

[CR36] Russell M, Rees G, Benton D, Kingsley M (2011). An exercise protocol that replicates soccer match-play. Int J Sports Med.

[CR37] Russell M, Benton D, Kingsley M (2012). Influence of carbohydrate supplementation on skill performance during a soccer match simulation. J Sci Med Sport.

[CR38] Russell M, Benton D, Kingsley M (2014). Carbohydrate ingestion before and during soccer match play and blood glucose and lactate concentrations. J Athl Train.

[CR39] Russell M, Sparkes W, Northeast J, Kilduff LP (2015). Responses to a 120 min reserve team soccer match: a case study focusing on the demands of extra-time. J Sport Sci.

[CR40] Russell M, West DJ, Bracken RM, Briggs MA, Giroud T, Cook CJ, Kilduff LP (2015). A passive heat maintenance strategy implemented during a simulated half-time improves lower body power output and repeated sprint ability in professional Rugby Union players. Plos One.

[CR41] Russell M, West DJ, Harper LD, Cook CJ, Kilduff LP (2015). Half-time strategies to enhance second-half performance in team-sports players: a review and recommendations. Sports Med.

[CR42] Smith TJ, Wilson MA, Karl JP (2016). Interstitial glucose concentrations and hypoglycemia during 2 days of caloric deficit and sustained exercise: a double-blind, placebo-controlled trial. J Appl Physiol (1985).

[CR43] Souglis A, Papapanagiotou A, Bogdanis GC, Travlos A, Apostolidis N, Geladas N (2015). Comparison of inflammatory responses to a soccer match between elite male and female players. J Strength Cond Res.

[CR44] Thomas DT, Erdman KA, Burke LM (2016). American College of Sports Medicine Joint Position Statement. Nutrition and athletic performance. Med Sci Sports Exerc.

