# Correction to: A comparison of isomaltulose versus maltodextrin ingestion during soccer-specific exercise

**DOI:** 10.1007/s00421-017-3750-6

**Published:** 2017-11-16

**Authors:** Emma J. Stevenson, Anthony Watson, Stephan Theis, Anja Holz, Liam D. Harper, Mark Russell

**Affiliations:** 1 Institute of Cellular Medicine, Newcastle University, Newcastle upon Tyne, UK; 2 Human Nutrition Research Centre, School of Agriculture, Food and Rural Development, Newcastle University, Newcastle upon Tyne, UK; 3 BENEO-Institute, Obrigheim/Pfalz, Wormserstrasse 11, Obrigheim, 67283 Germany; 4 Human and Health Sciences, University of Huddersfield, Huddersfield, UK; 5 School of Social and Health Sciences, Leeds Trinity University, Leeds, LS18 5HD UK

## Correction to: Eur J Appl Physiol (2017) 117:2321–2333 10.1007/s00421-017-3719-5

The article “A comparison of isomaltulose versus maltodextrin ingestion during soccer-specific exercise”, written by “Emma J. Stevenson, Anthony Watson, Stephan Theis, Anja Holz, Liam D. Harper, Mark Russell”, was originally published Online First without open access. After publication in volume [117], issue [11], page [2321–2333] the author decided to opt for Open Choice and to make the article an open access publication. Therefore, the copyright of the article has been changed to © The Author(s) [2017] and the article is forthwith distributed under the terms of the Creative Commons Attribution 4.0 International License (http://creativecommons.org/licenses/by/4.0/), which permits use, duplication, adaptation, distribution and reproduction in any medium or format, as long as you give appropriate credit to the original author(s) and the source, provide a link to the Creative Commons license, and indicate if changes were made. The original article was corrected.

